# Tick-borne encephalitis virus IgG antibody surveillance: vaccination- and infection-induced seroprevalences, south-western Germany, 2021

**DOI:** 10.2807/1560-7917.ES.2023.28.12.2200408

**Published:** 2023-03-23

**Authors:** Kathrin Euringer, Philipp Girl, Klaus Kaier, Jan Peilstöcker, Michael Schmidt, Michael Müller-Steinhardt, Beate Rauscher, Evelyn Bressau, Winfried V Kern, Gerhard Dobler, Johannes P Borde

**Affiliations:** 1 Bundeswehr Institute for Microbiology, National TBEV Consultant Laboratory, Munich, Germany; 2 Institute of Medical Biometry and Statistics (IMBI), University Medical Centre Freiburg, Faculty of Medicine, University of Freiburg, Freiburg i.Br., Germany; 3 DRK Blutspendedienst Baden-Württemberg – Hessen gGmbH, Frankfurt a.M., Germany; 4 Institut für Transfusionsmedizin und Immunhämatologie Baden-Baden, DRK Blutspendedienst Baden-Württemberg – Hessen gGmbH, Baden-Baden, Germany; 5 Gesundheitsamt des Ortenaukreises, Gengenbach, Germany; 6 Division of Infectious Diseases, Department of Medicine II, University Medical Centre Freiburg, Faculty of Medicine, University of Freiburg, Freiburg i.Br., Germany; 7 Praxis Prof. Borde & Kollegen, Oberkirch, Germany; 8 German Centre for Infection Research (DZIF), partner site Munich, Munich, Germany

**Keywords:** TBE, TBEV, NS1-ELISA, seroprevalence, Germany, tick-borne, vector-borne infections, viral infections

## Abstract

**Background:**

The exact epidemiology of tick-borne encephalitis virus (TBEV) infections is unknown because many TBEV infections have an influenza-like or asymptomatic course. Surveillance data are based on patients with any (predominantly neurological) symptoms that prompted diagnostic testing. Infection- and vaccine-induced antibodies against TBEV can be distinguished using an NS1 IgG ELISA.

**Aim:**

In a seroprevalence study we aimed to investigate TBEV antibody prevalence, incidences, manifestation indices and potential protection rates in a highly endemic district in south-western Germany.

**Methods:**

We analysed 2,220 samples from healthy blood donors collected between May and September 2021. The reported number of TBEV infections was provided on a sub-district level by the local public health authorities. Blood samples were first screened using a TBEV IgG ELISA. In a second step, all positive samples were further analysed with a recently established NS1 IgG ELISA. The presence of specific antibodies against TBEV (excluding cross-reacting antibodies against other flaviviruses) was confirmed by testing screening-positive samples with a microneutralisation assay.

**Results:**

Of 2,220 included samples, 1,257 (57%) tested positive by TBEV IgG ELISA and 125 tested positive for infection-induced TBEV NS1 antibodies, resulting in a TBEV NS1 IgG seroprevalence at 5.6% in our population. The yearly incidence based on the NS1 ELISA findings resulted in 283 cases per 100,000 inhabitants.

**Conclusion:**

Using the TBEV NS1 IgG assay, we confirmed a manifestation index of ca 2% and a high incidence of predominantly silent TBEV infections (> 250/100,000/year), which exceeds the incidence of notified cases (4.7/100,000/year) considerably.

**Key public health message**

**What did you want to address in this study?**

We wanted to find out how many infections with TBEV (tick-borne encephalitis virus) take place in an area in south-western Germany with high risk for this infection. Vaccinated and infected people both have TBEV antibodies. With a new test, we can distinguish between them and calculate the exact frequency of infection. Combined with officially notified TBEV infections, we can estimate the ratio of asymptomatic to symptomatic TBEV infections.

**What have we learnt from this study?**

We found that about one in 20 blood donors in a high-risk district acquired a TBEV infection. This figure is more than five-fold higher than in a similar study in non-vaccinated blood donors in the same district in 1986. Based on our findings only about 2% developed a symptomatic disease that prompted specific diagnostic and led to notification.

**What are the implications of your findings for public health?**

The risk of TBEV infection is much higher than previously assumed. In addition, the proportion of infected people developing severe infection seems to be smaller than estimated in earlier studies. This higher risk of infection implies changes in the environment affecting the virus’ transmission cycle and/or changes in human behaviour which increase the infection risk. We should ensure high vaccination rates for people who are exposed.

## Introduction

Tick-borne encephalitis virus (TBEV) is the most important tick-borne flavivirus on the Eurasian continent with 10,000–12,000 reported human cases per year [1]. There are three confirmed genetic subtypes, comprising the Far Eastern, European and Siberian subtype and at least two other subtypes (Baikalian, Himalayan) have recently been proposed, based on phylogenetic studies [2]. In central Europe, the only TBEV subtype detected to date is the European subtype [3]. Tick-borne encephalitis virus has been detected from Japan to France and the British Isles and is expanding its range more northern regions of Russia, Sweden and Finland [4]. Humans are only accidental hosts and get infected mainly by tick bite and rarely by alimentary infection with untreated milk or milk products from infected ungulates [2,3]. The occurrence of TBEV is closely associated with the presence of ticks. In Europe, *Ixodes ricinus* is the main vector of TBEV, whereas in the Far East and in Asia, *Ixodes persulcatus* is predominant [1]. 

In Germany, tick-borne encephalitis (TBE) became a notifiable disease in 2001. More than 7,600 TBE cases were notified between 2001 and 2021 (Robert Koch Institute, Survstat@rki [5]). TBE is a vaccine-preventable disease and national vaccination recommendations are issued by the Standing Committee on Vaccination (STIKO) at the RKI, where it is recommended to vaccinate against TBE all people who live or stay in a defined TBE risk area in Germany or in known foreign endemic areas and may have activities in nature [5]. Of note, the national public health authorities issued a general TBEV vaccination recommendation more than 20 years ago for southern Germany, notably the federal states of Bavaria and Baden-Wuerttemberg [6].

Manifestation of TBEV infection ranges from a subclinical course to clinically overt disease, with a variety of symptoms from febrile illness to severe neurological and even life-threatening or fatal central nervous system (CNS) symptoms [7]. According to current understanding of the disease, the ratio of clinical to subclinical forms and the proportion of patients with CNS symptoms are small. Epidemiological studies, dating back to the 1960s, indicate that ca 30% of TBEV infections manifest with unspecific influenza-like symptoms and about 10% of TBEV-infected develop CNS symptoms. These results are linked to a seroprevalence study in the area of Neunkirchen, Lower Austria, a highly endemic region at that time [8]. The authors correlated the seroprevalence in the population with the number of human cases diagnosed at a local hospital and calculated a manifestation index of 8.2% for CNS symptoms. Confirmatory studies have been hampered since the introduction of TBEV vaccines which have made it impossible to differentiate between vaccine-induced and infection-induced antibodies against TBEV infection. In addition, the emergence in central Europe of cross-reacting flaviviruses such as West Nile virus (WNV), Usutu virus (USUV), dengue virus (DENV), Zika virus (ZIKV), and travel-associated vaccinations against yellow fever virus (YFV) or Japanese encephalitis virus (JEV), may complicate sero-epidemiological surveys. Epidemiological studies conducted before the introduction of TBEV vaccines showed antibody prevalence in Germany ranging from 0.5% to 25% [9,10]. In occupationally exposed persons in Germany, seroprevalence was higher than in the common population with 3.3% up to 43% in forest workers, gardeners and farmers [11].

Recently, Girl et al. established an ELISA for the detection of antibodies against the non-structural protein 1 (NS1) of TBEV [12]. The idea was first presented by Albinsson et al., who proposed that NS1 might be a valuable tool in identifying TBE-infected among TBE-vaccinated people [13]. The NS1 protein of flaviviruses is produced during virus replication in cells and also secreted into the blood stream during TBEV infection, inducing antibody production in the infected person. Therefore, it is a reliable indicator of a current and past TBEV infection. Using this assay, infection-induced and vaccine-induced antibodies can be distinguished. Since the development of this ELISA, it has become technically feasible to conduct seroprevalence studies investigating infection rates.

We present seroprevalence data from a highly TBEV-endemic district in south-western Germany. The NS1 IgG ELISA was used in combination with a TBEV IgG ELISA and other serological tests to study the TBEV IgG prevalence in blood donors, to evaluate the proportion of TBEV-vaccinated and TBEV-infected persons in this population and to calculate the manifestation index of human TBEV infections. Similar antibody prevalence studies had been conducted in this region in 1964 and 1986 [9,10]. Therefore, a secondary aim was to provide evidence on the temporal development of the TBEV antibody prevalence.

## Methods

### Tick-borne encephalitis case definitions, manifestation index and incidence 

 Case definitions are issued by the Robert Koch Institute (RKI). The German case definition differs from the definition issued by the European Centre for Disease Prevention and Control (ECDC), as it includes also febrile forms of TBEV infections without CNS symptoms. The Robert-Koch Institute has since 2001 made the reported number of human TBEV infections available under open access in different formats, spatial and temporal resolutions [6].

The manifestation index (MI) in the region of interest (ROI) was calculated as a percentage:


$$MI\;(\%)=\frac{I\;(notified)}{I\;(NS1)}\times 100$$

The average notification-based incidence per 100,000 per year in the ROI was calculated on the basis of detailed 10-year national notification data:


$$I\;(notified)=\frac{a}{d}\times \frac{100,000}{10}$$

The average NS1 IgG-based incidence per 100,000 per year in the ROI was calculated on the basis of data on the NS1 IgG seroprevalence (spanning 20 years):


$$I\;(NS1)=\frac{b}{c}\times \frac{100,000}{20}$$

Whereby *a* represents the notified number of TBEV infections in the ROI spanning 10 years, *b* the number of NS1 IgG-positive samples in the ROI, *c* the number of all tested samples in the ROI and *d* the number of inhabitants in the ROI.

### Blood sample collection and sites of sampling

The collection of blood samples was conducted in collaboration with the regional blood transfusion/donation service (Deutsches Rotes Kreuz, DRK Blutspendedienst Baden-Wuerttemberg/Hessen gGmbH, Frankfurt a.M., Germany). Remainders of blood samples were used for further TBEV testing. Flyers summarising the study information were handed over to all voluntary blood donors at the donation site before the sampling began. Anonymised datasets for each sample included age, sex and postal code. Samples were collected between May and September 2021 in the Ortenaukreis, a county highly endemic for TBEV in the federal state of Baden-Wuerttemberg, south-western Germany. It is neighbouring France and has about 430,000 inhabitants [14]. In this rural Black-Forest region with three interspersed east–west river valleys, 408 TBE cases were notified between 2001 and 2021 [5]. For all calculations, we used a dataset provided by the local public health authorities. This dataset is spanning the time from 2011 to 2021 and is edited on a subdistrict/municipality level for our purposes.

### Enzyme-linked immunosorbent assays

**Figure 1:**
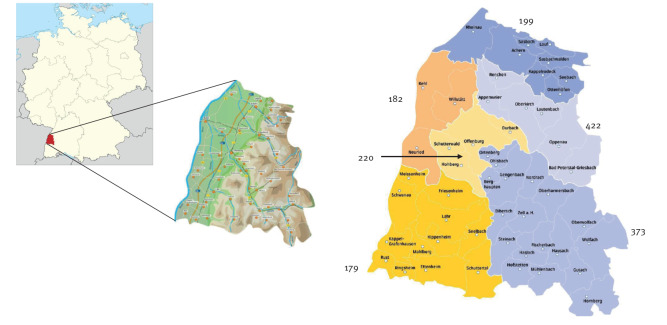
Study flowchart, Ortenaukreis, Germany, 2021 (n = 2,220)

DENV: dengue virus; ELISA: enzyme-linked immunosorbent assay; IgG: immunoglobulin G; IIFT: indirect immunofluorescence test; JEV: Japanese encephalitis virus; TBEV: tick-borne encephalitis virus; YFV: yellow fever virus.

For a comprehensive overview of the study, flow of samples and tests applied at each stage we refer to Figure 1. For the screening of IgG antibodies against TBEV, a TBEV IgG ELISA was performed using commercially available kits (Euroimmun AG, Lübeck, Germany). All plasma samples reacting positive in the screening ELISA were re-tested with an in-house TBEV NS1 IgG ELISA described previously [12]. Patient and control sera were tested in triplicate and interpreted as positive or negative with respect to their mean optical density as described [12].

### Microneutralisation assay

The microneutralisation assay (mNA) was conducted according to a standardised protocol which was adapted to TBEV [15]. We cultivated TBEV (strain Neudoerfl; kindly provided by Prof. F.X. Heinz, Institute of Virology, Medical University of Vienna, Austria) in A549 cells. The antibody titres corresponding to the highest serum dilution showing complete inhibition of cytopathic effect in both wells were reported. Samples were classified as either neutralisation titre (NT)-borderline (titre = 10), as negative (titre < 10) or NT-positive (titre ≥ 20). All borderline and negative samples were tested again by indirect immunofluorescence test (IIFT) to exclude that the ELISA results were cross-reactions with other flaviviruses infections or vaccinations.

### Indirect immunofluorescence test

Plasma with antibody titres of < 20 in the mNA were tested with a dilution of 1:10 in the indirect immunofluorescence assay using the IIFT Flavivirus Mosaik 1 (IgG) (TBEV, WNV, YFV, JEV) and the Mosaik dengue virus types 1–4 (IgG) (both Euroimmun). 

## Results

### Study population

**Figure 2:**
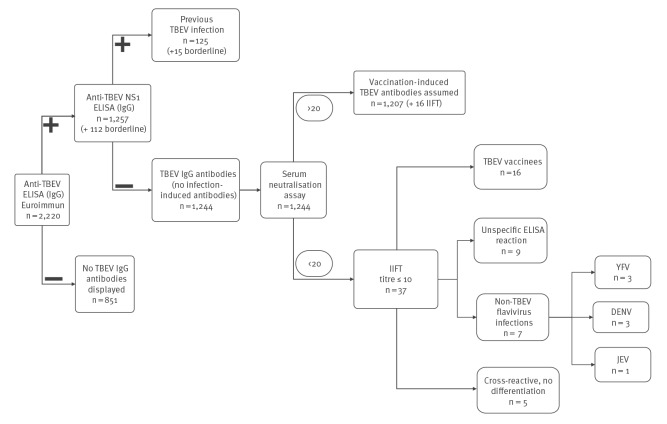
Age and sex distribution of the study population, blood donors, Ortenaukreis, Germany, May–September 2021 (n = 2,220)

Overall, 2,220 samples were included in the study. The mean age of the blood donors was 45.4 years (standard deviation: 15.4 years). See Figure 2 for age and sex distribution. There were 1,248 male donors and 972 female donors. The mean age of the male donors was 47.1 years (standard deviation: 15.0 years), the mean age of the female donors was 43.2 years (standard deviation: 15.8 years). The vast majority of the study population reported their place of residence within the district Ortenaukreis (1,824/2,220).

### TBEV IgG antibody seroprevalence

From the 2,220 included samples, 57% (1,257/2,220) tested positive using the screening TBEV IgG ELISA (Table). There were an additional 112 borderline results, which we categorised for calculating purposes in this first overview as negative. The mean age of the positive individuals was 47.2 years (standard deviation: 15.6 years). When excluding the borderline results, 38% of the samples were negative (851/2,220), 357 from female and 494 from male donors. The average age of the negative individuals was 48.4 years for males (n = 494; standard deviation: 15.2 years) and 45.5 years for females (n = 357; standard deviation: 16.1 years). From the 1,257 TBEV IgG-positive participants, 683 (54.7%) were male and 574 (59.1%) were female.

**Table:** Age-specific TBEV antibody seroprevalence based on IgG ELISA, Ortenaukreis, Germany, May–September 2021 (n = 2,200)

Age group (years)	Total	TBEV IgG-positive		TBEV IgG-negative		TBEV IgG-borderline		TBEV IgG-positive or borderline and					
								**NS1 IgG-positive**		**NS1 IgG-negative**		**NS1 IgG-borderline**	
%	n	%	n	%	n	%	n	%	n	%	n
18	23	70	16	30	7	0	0	4	1	65	15	0	0
19–24	281	64	180	33	94	2	7	9	24	57	160	1	3
25–29	266	63	167	34	91	3	8	8	21	56	148	2	6
30–34	191	58	110	39	74	4	7	5	10	56	107	0	0
35–39	106	60	64	35	37	5	5	7	7	58	62	0	0
40–44	154	63	97	29	45	8	12	6	9	64	99	1	1
45–49	162	58	94	35	56	7	12	5	8	60	97	1	1
50–54	311	56	173	40	123	5	15	6	19	54	168	0	1
55–59	308	48	148	44	136	8	24	4	11	51	158	1	3
60–64	217	56	122	39	85	5	10	3	6	58	126	0	0
** **≥ 65	201	43	86	51	103	6	12	4	9	44	89	0	0
**Total**	**2,220**	**57**	**1,257**	**38**	**851**	**5**	**112**	**6**	**125**	**55**	**1,229**	**1**	**15**

IgG: immunoglobulin G; NS1: non-structural protein 1; TBEV: tick-borne encephalitis virus.

### TBEV NS1 IgG seroprevalence

**Figure 3:**
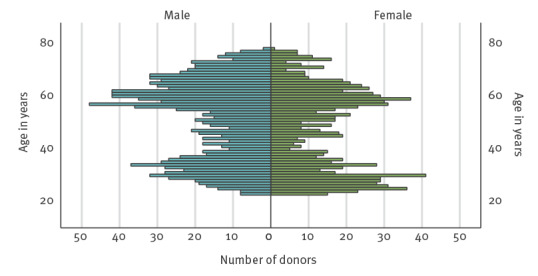
Seroprevalence of TBEV NS1 antibodies, by sex, blood donors, Ortenaukreis, Germany, May–September 2021 (n = 1,248)

IgG: immunoglobulin G; NS1: non-structural protein 1; TBEV: tick-borne encephalitis virus.

All samples that tested positive or borderline (n = 1,369) in the TBEV IgG ELISA were subsequently tested with the TBEV NS1 IgG ELISA to distinguish between vaccination-induced and infection-induced TBEV IgG antibodies. Of the overall initial 2,220 samples, 125 tested positive for infection-induced TBEV NS1 IgG antibodies, resulting in a TBEV NS1 IgG seroprevalence of 5.6% in the study population. Samples with borderline results were categorised as TBEV NS1 IgG-negative for calculating purposes (Table and Figure 3).

### Incidence 

**Figure 4:**
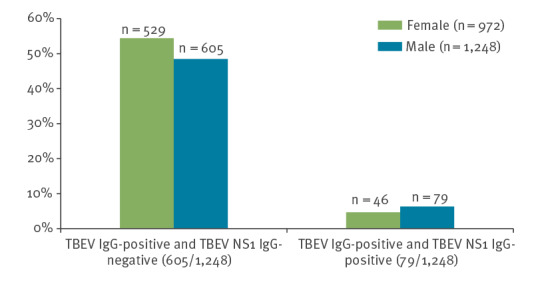
Calculated average TBEV incidence based on a positive TBEV NS1 IgG ELISA (per 100,000 inhabitants per year), regional data on a subdistrict scale, Ortenaukreis, Germany, extrapolated from data for May–September 2021 (n = 2,220)

NS1: non-structural protein 1; TBEV: tick-borne encephalitis virus.

Assuming that TBEV NS1 IgG can be detected for ca 20 years after TBEV infection (data not shown and [12]), the incidence could be calculated. We retrieved 408 notified TBEV infections from the SurvStat database of the RKI [5], spanning the period from 2001 to 2021. We calculated on this basis a yearly reported average incidence of 4.69 notified cases per 100,000 inhabitants. In order to calculate the real incidence based on NS1 ELISA data, we used the number of inhabitants recorded by the public authorities for each municipality in the district of interest. For comparison, the calculated yearly average incidence based on the NS1 ELISA findings (Figure 4) resulted in 283 infections per 100,000 inhabitants.

Numbers next to the subdistricts (differently coloured) of the district map indicate the calculated corresponding TBEV incidence based on a positive TBEV NS1 IgG ELISA (per 100,000 inhabitants per year).

### Manifestation index

We assumed that the TBEV infections notified during the period of interest were somehow symptomatic, because otherwise diagnostic procedures would not have been done. Therefore, we categorised them as symptomatic and calculated, using the NS1 IgG ELISA data (categorised as overall infections), an overall manifestation index of 2% in the studied high-endemic district.

### Spatial analysis of TBEV IgG antibody seroprevalence, TBEV NS1 IgG seroprevalence and manifestation index

For a more detailed analysis (appended as additional information in Supplementary Table S1), we subdivided the data on the basis of the geographical characteristics in the district of interest (Figure 4). As mentioned above, the district contains three interspersed east-west valleys (Achertal, Renchtal and Kinzigtal) and the Rhine River plain. The local public health authorities provided notification data spanning the years 2011 to 2021 on municipality level. 

The reported average incidence for the Achertal was five cases per 100,000 inhabitants per year. The calculated average incidence from our TBEV NS1 IgG data was 199 per 100,000 inhabitants per year. In the Renchtal, the notified incidence was nine per 100,000 inhabitants per year, whereas the incidence derived from our TBEV NS1 IgG data was 422 per 100,000 inhabitants per year. The reported average incidence for Kinzigtal was nine per 100,000 inhabitants per year, while the calculated average incidence from our TBEV NS1 IgG data was 373 per 100,000 inhabitants per year. The remaining sub-regions in the Ortenaukreis, Hanauerland, Offenburg and Lahr had a calculated average TBEV NS1 IgG incidence between 179 and 220 per 100,000 inhabitants per year – a detailed overview is included in Supplementary Table S1. It is striking that the reported TBE incidence in the Hanauerland area was overall low, with one per 100,000 inhabitants per year, compared with the TBEV NS1 IgG incidence in our analysis of 182 per 100,000 inhabitants per year. In this sub-region, the municipality of Neuried with no notified TBE cases, had a calculated average TBEV NS1 IgG incidence of 417 per 100,000 inhabitants per year. The dataset from that municipality included 36 blood donor samples, of which 27 were TBEV IgG-positive. Three samples were TBEV NS1 IgG-positive.

A further spatial analysis indicated higher incidences in the distant parts of the three valleys municipalities which might be hotspots for TBE infections. In detail, samples from Oberwolfach and Wolfach confirm, beside a high reported TBE incidence, a high TBEV NS1 IgG incidence reaching more than 700 per 100,000 inhabitants per year. In the neighbouring Renchtal the results from the distant villages Oppenau and Bad-Griesbach revealed similar high TBEV NS1 IgG incidence data.

### Microneutralisation assay and indirect immunofluorescence test

Anti-TBEV IgG antibodies were confirmed in 62% (1,369/2,220) of our tested samples (112 borderline samples included). The vast majority of the screening IgG-positive and NS1 IgG-negative samples showed neutralising antibody titres ≥ 20 in the mNA (97%; 1,207/1,244) and were therefore confirmed the IgG as TBEV-specific. The 37 samples that were classified as TBEV neutralisation antibody-negative (titre < 10) or TBEV neutralisation antibody-borderline (titre = 10) in the mNA were tested by IIFT and four could retrospectively be confirmed for TBEV IgG antibodies. Another 12 samples displayed a titre of 10 in the mNA and showed no flavivirus infection other than TBEV in the IIFT. These overall 16 donors were categorised as vaccinees, whose vaccination dated some time back, causing fading antibody titres. They were added to the 1,207 vaccinees whom we could confirm via mNA so that 98% (1,223/1,244) samples could ultimately be confirmed for TBEV-specific neutralising antibodies. Of the remaining 21 samples, nine are assumed to have reacted non-specifically in the Euroimmun screening ELISA, showing no mNA titre and no reaction in the IIFT. In addition, we found seven samples showing infections with flaviviruses other-than-TBEV in the IIFT, including three that showed antibodies against YFV, one that displayed antibodies against JEV and three against DENV. Finally, five samples could not be differentiated due to cross-reactivity in the IIFT.

## Discussion

Although TBE is a well-known disease in Europe, a number of epidemiological questions regarding the infection rate, the incidence and the manifestation index have remained unanswered for decades since the introduction of TBE vaccines, as differentiation between infection-induced and vaccine-induced antibodies in serological studies has been hampered. The present study approximates these unanswered questions of the exact TBEV infection incidence and manifestation index in a high-endemic district of southern Germany.

Using the recently established TBE NS1 IgG assay [12], the NS1 seroprevalence for the entire study sample indicated that 5.6% of the population had a past TBEV infection. This infection rate is in line with neighbouring countries such as Switzerland [16] or France [17] but is higher than in Lithuania [18]. Kaiser et al. detected an TBEV IgG seroprevalence of 9.4% in non-vaccinated people in south-western Germany. This finding is almost exactly the prevalence of 9.1% we found in our TBE IgG ELISA-positive group [19].

In Germany, the first TBEV vaccine was licensed in 1981. In the 1980s, highly exposed population groups like forest workers were vaccinated with priority. In 1986, still before the general national recommendations for TBEV vaccination in high-risk regions, Ackermann et al. conducted a TBEV seroprevalence study in blood donors of the same district as investigated in our study – the Ortenaukreis, Federal State of Baden-Wuerttemberg [10]. Neutralising antibodies against TBEV were found in 0.8% of the samples. Comparing our results with these historical data, infection-induced TBEV seroprevalence has increased ca 7-fold in this particular district over the past 40 years.

Our study confirmed a manifestation index of ca 2% and a high incidence of predominantly asymptomatic TBEV infections (> 250/100,000), which exceeded the incidence of notified cases (4.7/100,000) by magnitudes. It should be kept in mind that TBEV case definitions differ within the European Union. The ECDC defines TBEV cases as occurrence of CNS symptoms in addition to a confirmed laboratory result, while in Germany, influenza-like or even asymptomatic disease meets the TBEV case definition issued by the RKI. The lack of a uniform case definition needs to be taken into consideration when comparing our calculated values with previous studies which may have used other definitions than in our study. However, our setting including all TBE cases, irrespective of the clinical symptoms, better approximates the true biological and epidemiological situation as if only severe clinical cases are included.

Our findings suggest that the immune protection rate (the proportion of TBEV neutralisation antibody-positive study participants) in the study population might be higher than reported in previous studies evaluating TBEV vaccination rates [20]. In the screening ELISA, 62% of our tested samples displayed TBEV IgG antibodies. For 98%, the IgG-positive and NS1-negative samples could be confirmed using the mNA assay. Our study population consisted of blood donors, who are considered to have a higher awareness of health-associated topics such as vaccinations and preventive medicine than the average population. Therefore, a direct comparison to the general population should be made only cautiously. Notably, the age of our participants ranged from 18 to 72 years, excluding children and a part of the elderly population.

Tick-borne encephalitis virus occurs in well-defined areas, so called TBEV microfoci. The five-fold higher number of TBEV infections compared with blood donors from 1986 could be explained by different biotic, abiotic and anthropogenic factors [9]. Increased tick activity following changing climate conditions could be a possible reason for increased infection rates. A study from Sweden illustrates that disease incidence was correlated with mild winters and early arrival of spring [21]. Notably, increasing host animal populations as well as more inhabitants around TBEV foci could contribute to higher TBEV incidences. However, in our district of interest, the rural structure of the valleys and population densities has been roughly constant over the past decades. Increased notification and awareness, especially in the local primary care system, could be a crucial factor for the rise in case numbers as well.

When comparing the six subregions in the Ortenaukreis, TBEV infection rates were clustered in specific areas. The incidences were particularly high in the three valleys (199/100,000 in Achertal, 422/100,000 in Renchtal and 373/100,000 in Kinzigtal), whereas they were lower in the Rhine River plane. The reason is probably the natural spread of TBEV microfoci in nature along geographical structures such as rivers or valleys. The distribution of these microfoci in a landscape is linked to local climate and microclimate conditions. The TBEV infection rates were especially high in Neuried, an area close to the Rhine River. There were no notified cases in this municipality. We hypothesise that there might be a nearby undiscovered natural TBEV microfocus in the tick population or that anthropogenic factors (e.g. high intermittent migration/travelling into highly endemic areas) might be responsible.

Our study has several limitations. There might be a certain bias in the composition of our sample population as shown in the population diagram. The age distribution suggests a gap regarding middle-aged blood donors. This biased age distribution might have led to a manifestation index calculated too low, as it is known that disease severity increases with age. However, as shown in the age distribution of human cases in Germany, the case numbers increase especially in those 50 years and older [22]. Therefore, the bias may be negligible. When discussing the present results, it should be kept in mind that the population sample analysed may not be representative for other regions in Germany because of different biotic, abiotic and anthropogenic factors.

To date, it is not clear if TBE vaccination induces sterile immunity in humans. Therefore, we cannot estimate whether or not the protected 62% would have produced an NS1 antibody response after infection. This question might, however, influence our manifestation index. In a model using vaccinated mice, virus replication took place [23]. If the vaccination in humans results in protection but not sterile immunity, a number of TBEV NS1 IgG-positive blood donors may have been infected after vaccination and produced serological evidence of infection in the absence of clinical disease. This might have led to a much smaller manifestation index as seen under natural non-vaccinated conditions. However, we have so far not been able to distinguish between pre- or post-vaccination infections.

The broad recommendation of TBEV vaccinations impacts the overall occurrence of TBEV infections. Vaccination failures, resulting in TBEV breakthrough infections, are rare – ranging from 1.7% to 6.7% of the reported TBEV infection cases in Germany [24]. The current situation with an estimated vaccination rate of 30–40% in the population is not comparable to the setting of a de facto unvaccinated population in older epidemiological studies [9,10]. This results in an underestimation of the manifestation index for TBEV infections which is often cited in the literature as ca 10%, which dates back to a study of 1965 [8].

In this study, we used the Euroimmun TBEV IgG ELISA for screening. Since the ELISA method in general is very sensitive, yet not as specific, we confirmed all screening ELISA-positive and NS1 IgG ELISA-negative samples by mNA. We assume that samples without detectable neutralising capacity constitute vaccination failures, vaccinations given some time ago or cross-reactivity with a non-TBEV flavivirus infection. A negligible number of our samples tested positive for other flaviviruses (0.06%), e.g. DENV and YFV. These viruses are not endemic in Europe and are considered to be travel-related. While we did not review our participants’ vaccination status against YFV and JEV, it is known that these viruses induce of cross-reactive antibodies against TBEV [25]. The rate of other flavivirus vaccinations and/or infections may be higher than observed in this study as the primary inclusion criterion was the presence of TBEV IgG antibodies. Therefore, we have missed all sera which reacted mono-specifically against the tested flaviviruses without cross-reactions against TBEV. These limitations need to be considered when interpreting the results. A further critical point is the inability of the NS1 IgG assay to detect past (> 20 years) TBEV infections due to fading antibodies. The 20-year threshold was taken into account when calculating incidences, however, this threshold was chosen somewhat arbitrarily as the evidence for this approach was based on a few cases of TBEV infection with persistent antibodies after about 20 years [12].

## Conclusion

Our study contributes to our understanding of theTBEV infection incidence and manifestation index in a high-endemic district of southern Germany. Notably, there seems to be an increase in TBEV infection rates, as shown for the Ortenaukreis. We present a comprehensive update on the seroprevalence and incidences, which indicate a rise in TBEV infections in the last decades in the district Ortenaukreis.

## Supplementary Data

367000 Supplement
