# Supplementary material for: Tick-borne encephalitis virus IgG antibody surveillance: vaccination- and infection-induced seroprevalences, south-western Germany, 2021
Source: Euro Surveill. 2023 Mar 23;28(12):2200408. doi: 10.2807/1560-7917.ES.2023.28.12.2200408 (PMC10037664; doi:10.2807/1560-7917.ES.2023.28.12.2200408)
Supplement: Supplement [file 22-00408_BORDE_Supplement.pdf]

This supplementary material is hosted by Eurosurveillance as supporting information alongside the article [*Tick-borne encephalitis virus IgG antibody surveillance: vaccination- and infection-induced seroprevalences, south-western Germany, 2021*] on behalf of the authors who remain responsible for the accuracy and appropriateness of the content. The same standards for ethics, copyright, attributions and permissions as for the article apply. Supplements are not edited by Eurosurveillance and the journal is not responsible for the maintenance of any links or email addresses provided therein.

Suppl. Table 1

Achertal	Gemeinden	Einwohner	Meldefälle/ 10 Jahre	Meldeinzidenz 100.000 Einwohner / Jahr	Alle Spender	FSME IgG NEGATIV in 20 Jahre	FSME IgG Positiv in 20 Jahre	NS1 Positiv in 20 Jahre	errechnete Inzidenz aus NS1 Daten je 100.000 Einwohner/Jahr	Manifestationsindex	Schutzrate (quasi-Impfrate) in %
	Achern	25797	10	4	294	170	124	9	153	3%	39%
	Kappelrodeck	6164	3	5	41	20	21	1	122	4%	49%
	Ottenhöfen	3205	4	12	52	25	27	2	192	6%	48%
	Lauf	4015	3	7	10	4	6	2	1000	1%	40%
	Sasbach	5277	4	8	17	9	8	1	294	3%	41%
	Sasbachwalden	2594	2	8	12	3	9	1	417	2%	67%
	Rheinau	11295	1	1	8	6	2	0	0	n.d.	25%
	Seebach	1434	2	14	19	11	8	2	526	3%	32%
	Summe	59781	29	5	453	248	205	18	199	2%	41%
Renchtal	Gemeinden	Einwohner	Meldefälle/ 10 Jahre	Meldeinzidenz 100.000 Einwohner / Jahr	Alle Spender	FSME IgG NEGATIV in 20 Jahre	FSME IgG Positiv in 20 Jahre	NS1 Positiv in 20 Jahre	errechnete Inzidenz aus NS1 Daten je 100.000 Einwohner/Jahr	Manifestationsindex	Schutzrate (quasi-Impfrate) in %
	Appenweier	10240	2	2	19	6	13	2	526	0%	58%
	Renchen	7421	1	1	154	64	90	10	325	0%	52%
	Oberkirch	19913	14	7	45	21	24	4	444	2%	44%
	Lautenbach	1930	3	16	1	1	0	0	0	n.d.	0%
	Oppenau	4844	17	35	44	15	29	6	682	5%	52%
	Bad Peterstal-Griesbach	2748	7	25	45	21	24	4	444	6%	44%
	Summe	47096	44	9	308	128	180	26	422	2%	50%
Kinzigtal	Gemeinden	Einwohner	Meldefälle/ 10 Jahre	Meldeinzidenz 100.000 Einwohner / Jahr	Alle Spender	FSME IgG NEGATIV in 20 Jahre	FSME IgG Positiv in 20 Jahre	NS1 Positiv in 20 Jahre	errechnete Inzidenz aus NS1 Daten je 100.000 Einwohner/Jahr	Manifestationsindex	Schutzrate (quasi-Impfrate) in %
	Ortenberg	3422	0	0	25	7	18	3	600	0%	60%
	Steinach	3945	3	8	2	0	2	0	0	n.d.	100%
	Mühlenbach	1688	1	6	1	1	0	0	0	n.d.	0%
	Ohlsbach	3325	1	3	97	41	56	6	309	1%	52%
	Berghaupten	2451	0	0	54	23	31	3	278	0%	52%
	Biberach	3732	2	5	3	2	1	0	0	n.d.	33%
	Fischerbach	1767	1	6	1	0	1	0	0	n.d.	100%
	Gengenbach	10976	14	13	121	57	64	7	289	4%	47%
	Haslach i.K.	7154	4	6	1	0	1	0	0	n.d.	100%
	Nordrach	1855	6	32	1	1	0	0	0	n.d.	0%
	Oberwolfach	2585	5	19	35	9	26	5	714	3%	60%
	Wolfach	5691	9	16	46	7	39	5	543	3%	74%
	Hausach	5743	5	9	13	0	13	1	385	2%	92%
	Zell a.H.	8129	5	6	2	2	0	0	0	n.d.	0%
	Summe	62463	56	9	402	150	252	30	373	2%	55%
Hanauerland	Gemeinden	Einwohner	Meldefälle/ 10 Jahre	Meldeinzidenz 100.000 Einwohner / Jahr	Alle Spender	FSME IgG NEGATIV in 20 Jahre	FSME IgG Positiv in 20 Jahre	NS1 Positiv in 20 Jahre	errechnete Inzidenz aus NS1 Daten je 100.000 Einwohner/Jahr	Manifestationsindex	Schutzrate (quasi-Impfrate) in %
	Kehl	37358	6	2	67	37	30	1	75	2%	43%
	Willstätt	9962	1	1	7	0	7	0	0	n.d.	100%
	Neuried	9856	0	0	36	9	27	3	417	0%	67%
	Summe	57176	7	1	110	46	64	4	182	1%	55%
Offenburg	Gemeinden	Einwohner	Meldefälle/ 10 Jahre	Meldeinzidenz 100.000 Einwohner / Jahr	Alle Spender	FSME IgG NEGATIV in 20 Jahre	FSME IgG Positiv in 20 Jahre	NS1 Positiv in 20 Jahre	errechnete Inzidenz aus NS1 Daten je 100.000 Einwohner/Jahr	Manifestationsindex	Schutzrate (quasi-Impfrate) in %
	Offenburg	60824	20	3	123	51	72	9	366	1%	51%
	Hohberg	8203	2	2	42	20	22	1	119	2%	50%
	Durbach	3991	2	5	8	4	4	0	0	n.d.	50%
	Schutterwald	7227	2	3	100	48	52	2	100	3%	50%
	Summe	80245	26	3	273	123	150	12	220	1%	51%
Altkreis Lahr	Gemeinden	Einwohner	Meldefälle/ 10 Jahre	Meldeinzidenz 100.000 Einwohner / Jahr	Alle Spender	FSME IgG NEGATIV in 20 Jahre	FSME IgG Positiv in 20 Jahre	NS1 Positiv in 20 Jahre	errechnete Inzidenz aus NS1 Daten je 100.000 Einwohner/Jahr	Manifestationsindex	Schutzrate (quasi-Impfrate) in %
	Ringsheim	2444	1	4	5	1	4	0	0	n.d.	80%
	Meissenheim	4056	0	0	59	24	35	1	85	0%	58%
	Kappel-Grafenhausen	5190	0	0	7	2	5	1	714	0%	57%
	Friesenheim	13452	2	1	129	67	62	5	194	1%	44%
	Schuttertal	3173	4	13	1	1	0	0	0	n.d.	0%
	Mahlberg	5310	0	0	5	1	4	0	0	n.d.	80%
	Ettenheim	13595	1	1	77	24	53	3	195	0%	65%
	Kippenheim	5605	3	5	5	1	4	0	0	n.d.	80%
	Lahr	47887	9	2	94	38	56	4	213	1%	55%
	Rust	4481	0	0	3	2	1	0	0	n.d.	33%
	Seelbach	4953	4	8	6	1	5	0	0	n.d.	83%
	Summe	110146	24	2	391	162	229	14	179	1%	55%
